# Versatility of Pyridoxal Phosphate as a Coating of Iron Oxide Nanoparticles

**DOI:** 10.3390/nano7080202

**Published:** 2017-07-29

**Authors:** Debora Bonvin, Ulrich J. Aschauer, Jessica A. M. Bastiaansen, Matthias Stuber, Heinrich Hofmann, Marijana Mionić Ebersold

**Affiliations:** 1 Powder Technology Laboratory, Institute of Materials, Ecole Polytechnique Fédérale de Lausanne, Lausanne 1015, Switzerland; debora.bonvin@epfl.ch (D.B.); heinrich.hofmann@epfl.ch (H.H.); 2 Department of Chemistry and Biochemistry, University of Bern, Bern 3012, Switzerland; ulrich.aschauer@dcb.unibe.ch; 3 Department of Radiology, University Hospital (CHUV) and University of Lausanne (UNIL), Lausanne 1011, Switzerland; jbastiaansen.mri@gmail.com (J.A.M.B.); Matthias.Stuber@chuv.ch (M.S.); 4 Center of Biomedical Imaging (CIBM), Lausanne 1011, Switzerland

**Keywords:** iron oxide nanoparticles, cellular uptake, magnetic resonance imaging, pyridoxal 5′-phosphate, surface functionalization

## Abstract

Pyridoxal 5′-phosphate (PLP) is the most important cofactor of vitamin B_6_-dependent enzymes, which catalyses a wide range of essential body functions (e.g., metabolism) that could be exploited to specifically target highly metabolic cells, such as tumour metastatic cells. However, the use of PLP as a simultaneous coating and targeting molecule, which at once provides colloidal stability and specific biological effects has not been exploited so far. Therefore, in this work iron oxide nanoparticles (IONPs) were coated by PLP at two different pH values to tune PLP bonding (e.g., orientation) at the IONP surface. The surface study, as well as calculations, confirmed different PLP bonding to the IONP surface at these two pH values. Moreover, the obtained PLP-IONPs showed different zeta potential, hydrodynamic radius and agglomeration state, and consequently different uptake by two metastatic-prostate-cancer cell lines (LnCaP and PC3). In LnCaP cells, PLP modified the morphology of IONP-containing intracellular vesicles, while in PC3 cells PLP impacted the amount of IONPs taken up by cells. Moreover, PLP-IONPs displayed high magnetic resonance imaging (MRI) *r*_2_ relaxivity and were not toxic for the two studied cell lines, rendering PLP promising for biomedical applications. We here report the use of PLP simultaneously as a coating and targeting molecule, directly bound to the IONP surface, with the additional high potential for MRI detection.

## 1. Introduction

The biomedical use of nanoparticles (NPs) strongly depends on their colloidal stability in physiological conditions. Their stabilization in aqueous medium is achieved by coating NPs with various biocompatible molecules. Large polymers (e.g., polyethylene glycol, PEG) or sugars (e.g., dextran, chitosan) are among the most commonly used coatings for the steric stabilization of NPs [1]. However, the final hydrodynamic diameter (*d_H_*) of such NPs tends to be very large (typically up to hundreds of nanometers), especially after the addition of targeting moieties or drugs. Therefore, charged molecules, which provide electrostatic stabilization, are often preferred, because they are small and therefore allow the maintenance of an overall diameter of NPs that is as small as possible. This small size is highly needed to overcome specific biological barriers, for instance to reach lymph node metastases, or to increase the amount of NPs inside small volumes (e.g., metastases) for increased therapeutic efficacy and/or imaging capability. The coating not only determines the size of the NPs, but also, via specific chemical groups and their charge, the affinity of the coating to the NPs’ surface, and the possibility to further couple the NPs with targeting molecules and/or drugs. Consequently, the choice of the coating molecule is crucial, not only because it determines the surface properties of the NPs (chemical groups, size, charge), but also because in turn it directly affects the biological behaviour of NPs, especially their cellular uptake [2], biodistribution [3], blood circulation [4] and metabolism [5]. Among these biological effects, the biodistribution of NPs has received particular attention. Coupling the NPs with a targeting agent in order to specifically deliver NPs to body regions, such as tumours, dramatically enhances their clinical utility [6,7,8]. Various approaches have been reported for the active targeting of NPs, especially those which use antibodies, aptamers and smaller molecules. In particular, the use of small targeting molecules, or even the use of one molecule as a simultaneously targeting and coating molecule, would significantly decrease the overall size of NPs. Unfortunately, this combination is so far not fully exploited.

Among the numerous small bio-molecules, pyridoxal 5′-phosphate (PLP), which is the phosphorylated form of pyridoxal found in vitamin B_6_, is a very promising molecule to coat and target NPs, because among the molecules present in vitamin B_6_, PLP has the most impact in the body (it is the cofactor of most vitamin B_6_-dependent enzymes, catalysing a large range of biochemical reactions) [9]. PLP-dependent enzymes are especially involved in essential cellular processes, as for instance in the biosynthesis of amino acids or neurotransmitters, in the synthesis of heme, or in the regulation of important genes (e.g., glucocorticoid hormones, albumin) [10]. PLP has therefore an effect on the regulation of essential body functions, especially cardiovascular, metabolic, immunologic and homeostatic functions. For all these reasons, it is of interest to use PLP as a coating of NPs, especially of iron oxide NPs (IONPs), which are extensively studied for their use as medical diagnostic and therapeutic agents.

**Figure 1:**
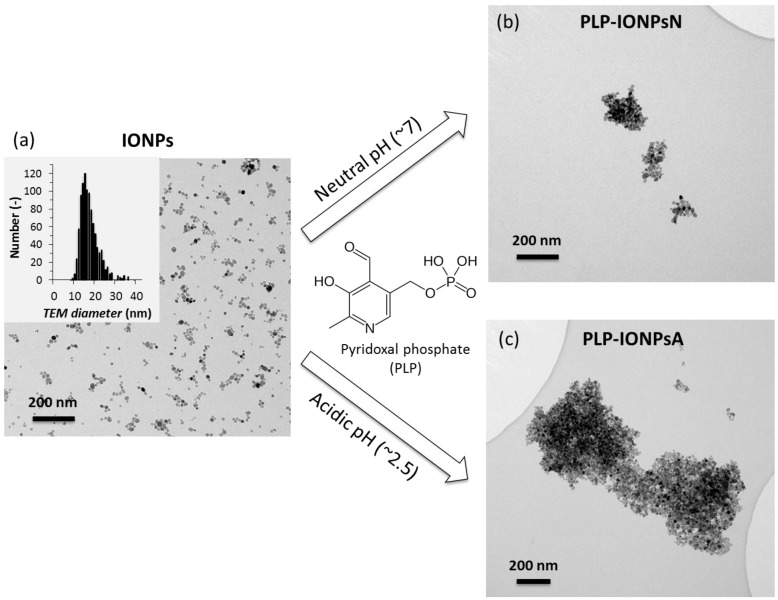
Representative TEM micrographs of uncoated iron oxide nanoparticles (IONPs) (**a**), but also of IONPs coated by pyridoxal phosphate (PLP) at neutral pH (PLP-IONPsN) (**b**) or at acidic pH (PLP-IONPsA) (**c**). The insert in (**a**) shows the distribution of the TEM diameter of uncoated IONPs. The chemical structure of PLP is given in the middle of the figure.

PLP is not only biocompatible, but it has also four chemical groups (phosphoryl, pyridinium nitrogen, phenolic and aldehyde groups, see the chemical structure of PLP in Figure 1) which render this molecule highly bio-active [11]. This versatility of chemical groups has been exploited to bind PLP on the surface of various types of NPs. Usually, the phosphate group has been used to bind PLP to the NP surface, while the aldehyde and/or phenolic groups have been employed to couple the PLP-coated NPs with anti-cancer drugs. In other words PLP is typically playing the role of a link between the NP and the active molecule [12]. Apart from their use as linking groups, the former two chemical groups have also been used to chelate metal ions, especially at the surface of gold NPs [13], silica NPs, oxidized carbon nanotubes or silica-coated IONPs [14]. Therefore, PLP has been mainly used as a linker and chelating agent. However, various chemical groups of PLP—if in the deprotonated state, i.e., charged—could be used to electrostatically stabilize NPs and could therefore in the suitable coating conditions enable the use of PLP as a coating molecule. Furthermore, given that PLP plays the essential and very specific functional roles in the human body given above, PLP could be bound to the surface of NPs to trigger specific biological effects and to give NPs interesting features. For example, the importance of PLP in the metabolism could be exploited to specifically target NPs to highly metabolic cells, such as cancer cells and especially metastatic cells. Unfortunately, both the coating of NPs with PLP to stabilize them and their use as a targeting agent has not been studied so far.

In the present study, we coated IONPs with PLP using a one-step coating reaction at two different pH values, 7.0 and 2.5, in order to differently orient the PLP molecule at the IONPs’ surface, and thus tune the behaviour of IONPs that are in contact with biological matter, while preserving the targeting activity of PLP bound to the IONPs’ surface. In order to study the influence of the PLP orientation at the IONPs’ surface, the surface of PLP-coated IONPs was characterized by spectroscopy (Fourier transform infrared spectroscopy (FTIR), X-ray photoelectron spectroscopy (XPS) and UV-visible spectroscopy). In order to evaluate the potential use of PLP as a coating molecule, we measured the zeta potential and studied the agglomeration state of PLP-coated IONPs by transmission electron microscopy (TEM) and by dynamic light scattering (DLS) in both water and two biological media. We also measured the magnetic resonance imaging (MRI) relaxivity of PLP-IONPs to evaluate their potential for diagnostics by MRI. Moreover, we investigated their cellular uptake by two metastatic-prostate-cancer cell lines (LnCaP and PC3) and their toxicity in order to explore the possibility of using PLP on the IONP’s surface as a coating molecule and simultaneously as a targeting agent for controlling the biological behaviour of PLP-IONPs.

## 2. Results and Discussion

### 2.1. Binding of PLP to IONPs

IONPs were synthesized by our previously developed method, combining the co-precipitation (CP) method and hydrothermal (HT) treatment, referred to as CP + HT synthesis [15,16], which was modified for this study. The TEM micrograph and size distribution of the uncoated IONPs (mean TEM diameter 16.7 ± 4.5 nm) are given in Figure 1a. These IONPs were then coated with PLP (given in Figure 1), which was reported to have dissociation constants of <2.50, 4.14, 6.2 and 8.69 for the first dissociation of the phosphoryl group, for the phenolic group, for the second dissociation of the phosphoryl group, and for the pyridinium nitrogen, respectively [17,18]. In order to tune the interaction of PLP and the surface of IONPs, we took the advantage of the difference in both the surface charges of IONPs and the protonation state of PLP at different pH values. Therefore, we performed the coating process at two different pH values, neutral (pH 7.0) and acidic (pH 2.5), and the samples obtained in this way were denoted PLP-IONPsN and PLP-IONPsA, respectively.

**Figure 2:**
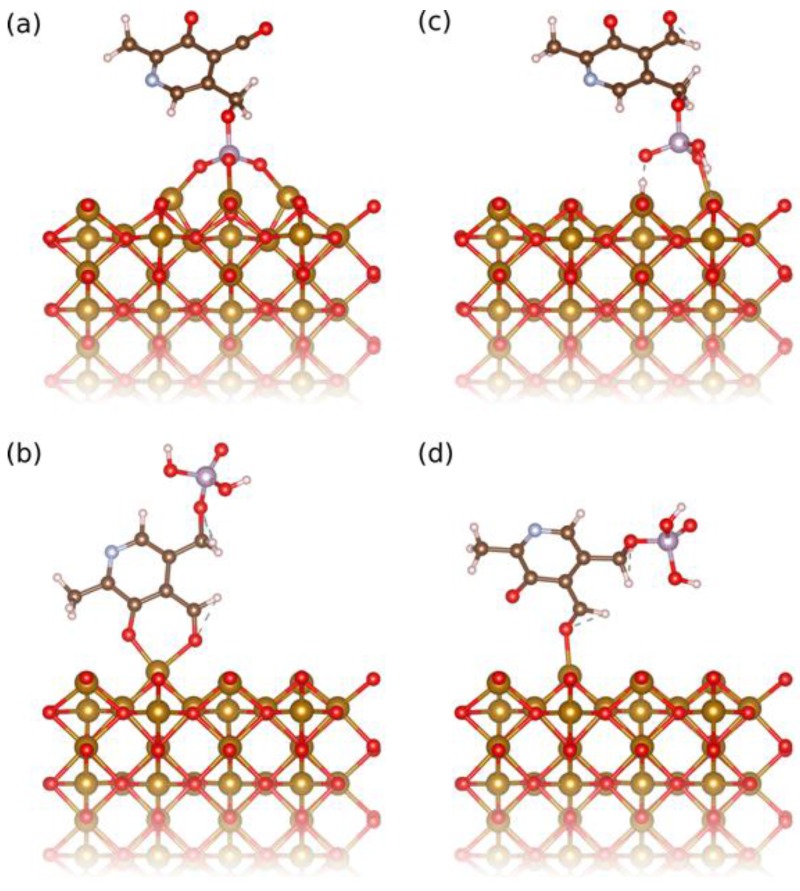
Relaxed adsorption configuration of (**a**) the deprotonated phosphoryl group, (**b**) the deprotonated phenyl group bidentate with the aldehyde group, (**c**) the protonated phosphoryl and (**d**) aldehyde groups of PLP on the (110) surface of iron oxide used to compute the binding energies in the studied cases.

At pH 7.0, the surface of IONPs is slightly positively charged (zeta potential, *ξ*, is about +5 mV), while the deprotonated phenolic and phosphoryl groups are negatively charged and thus, in this condition the surface of IONPs can be functionalized by PLP due to the electrostatic interactions of the opposite charges. The deprotonated phosphoryl group was found by density functional theory (DFT) calculations to have a binding energy of 3.54 eV (Figure 2a), while the deprotonated phenolic group adsorbs in a bidentate fashion together with the neighboring aldehyde group with 1.94 eV (Figure 2b). Attempts to adsorb only the phenolic group were unsuccessful, however the aldehyde group could be adsorbed separately and has a binding energy of 1.00 eV (Figure 2d). Assuming that the binding energies are additive, this means that the phenolic group alone contributes about 0.94 eV binding energy. All these suggest stronger bonding of the deprotonated phosphoryl group, which involves all phosphoryl terminations, leading to multidentate bonding (Figure 2a). In addition, the adsorption of the pyridinium nitrogen on the surface of IONPs could not be computed, because the deprotonated phosphoryl group always adsorbs first. However, in the other coating condition, at pH 2.5, the surface of IONP is strongly charged (*ξ* is about +50 mV) and thus able to attract: (A) the partially deprotonated phosphoryl group (Figure 2c), but also (B) protonated phenolic and aldehyde groups that absorb together as bidentate in a configuration very similar to the ones of the bidendate deprotonated phenolic and aldehyde groups shown in Figure 2b. The bonding energies of these two groups, (A) and (B), were found by DFT calculations to be more comparable (1.74 eV and 0.82 eV, respectively) than at pH 7.0. Thus, at both coating pH values, the bonding of PLP to the surface of the IONPs occurs by preferential adsorption of the phosphoryl group. However, the phosphoryl group bonding is much more favorable at pH 7.0 than at pH 2.5. This suggests that the bonding of PLP to the IONPs’ surface proceeded more via the fully deprotonated phosphoryl group at pH 7.0, and via both partially deprotonated phosphoryl and aldehyde groups at pH 2.5.

### 2.2. Surface Characterization

The surface of the uncoated and both PLP-coated IONPs (PLP-IONPsN and PLP-IONPsA) were studied by XPS and the obtained elemental composition (Fe, O, C, N and P) of these samples is given in Table S1. The presence of PLP on the surface of IONPs in both coated samples is indicated by the detection of P 2p XPS peaks (1.1 at% and 1.3 at% of P were found in PLP-IONPsN and PLP-IONPsA, respectively) in coated as compared to uncoated IONPs (Table S1). The XPS spectra, overall (a) and the Fe 2p, N 1s, C 1s and P 2p peaks (b to e), of uncoated and coated IONPs are shown in Figure S1. For all samples, the Fe 2p_3/2_ was found at 710 eV (Figure S1b), which is in agreement with previous reports. [19] Moreover, the bonding energy of the P 2p bond is considered as a good indicator of the attachment of the phosphoryl group, since deprotonation of one or both P–OH terminals can be easily seen by P 2p shifts to 133.6 eV and 133.2 eV, respectively [19,20]. Indeed, the P 2p bonding energy for the fully deprotonated PLP in the sample PLP-IONPsN was found at 133.28 eV (Figure S1e), while for PLP-IONPsA it was found at 133.5 eV. The last value is higher than for the PLP-IONPsN sample, but still lower than for the protonated PLP (134.1 eV), suggesting that PLP is partially deprotonated due to the possibility for PLP to form intermolecular bonding between molecules on the surface of either the same IONP or between different IONPs [20].

**Figure 3:**
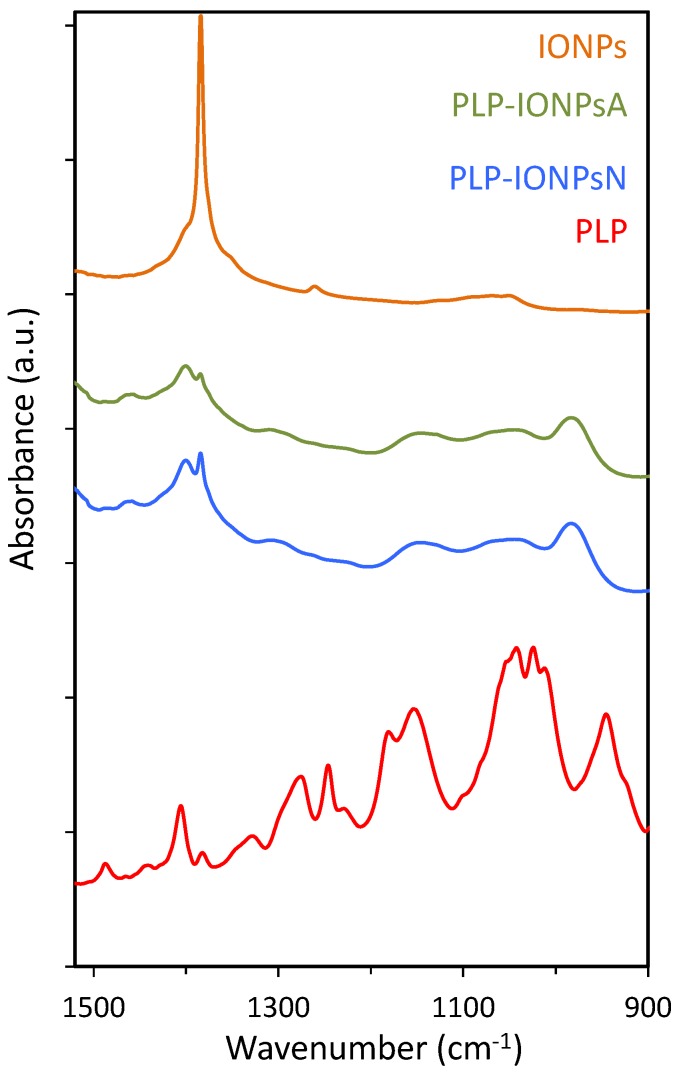
The FTIR absorbance spectra of uncoated IONPs, PLP-IONPsA, PLP-IONPsN and PLP.

Additional information on the attachment of PLP to the surface of IONPs was obtained by FTIR (Figure 3). In particular, the FTIR spectra in the 900–1500 cm^−1^ region, containing the characteristic P=O and P–O stretching vibrations, is given in Figure 3. As expected, the characteristic peaks at 1383 cm^−1^, which is typical for the γ-Fe_2_O_3_ composition of IONPs [16], were observed in all samples. Also, the characteristic P=O and P–O–H bands around 1250 and 920 cm^−1^, respectively, were not observed in the coated samples. Instead, a broad band from ~950 to ~1200 cm^−1^ was found in both coated samples and this is typically associated with multidentate bonding [21], which is in agreement with the above given XPS results.

The influence of PLP on the surface properties of IONPs was assessed by measuring the zeta potential (*ξ*) of uncoated and PLP-coated IONPs. The *ξ* values were firstly measured in water, which allowed us to assess the effect of the difference in PLP coating on the surface properties of two PLP-coated IONPs without the interference of proteins. As expected, we found that the *ξ* in water changed between uncoated and PLP-coated IONPs, confirming that the IONPs’ surface is coated with PLP molecules (Table 1). In addition, a difference in *ξ* was observed between PLP-IONPsN and PLP-IONPsA, confirming that the binding of PLP to the surface of IONPs is different between the two types of PLP-coated IONPs. More precisely, the *ξ* of PLP-IONPsN was more negative than that of PLP-IONPsA. This is in agreement with the above given results, especially with the results of bonding calculations, which suggested that PLP preferentially bonds to the surface of IONPs via the deprotonated phosphoryl group at pH 7, but via both protonated phosphoryl and phenolic groups at pH 2.5, leading to more negative *ξ* in the first case.

For possible biomedical applications, the characterization of IONPs in medium, i.e., in the presence of proteins, is crucial, since proteins present in the biological environment can change the surface and the *ξ* of NPs [22]. The *ξ* was therefore measured in Roswell Park Memorial Institute (RPMI) medium and Dulbecco’s Modified Eagle Medium (DMEM; Table 1), which were the media used in the cell experiments performed in the present study. We found that the *ξ* in the media were different from the *ξ* measured in water, confirming that proteins covered the IONPs’ surfaces as expected and modified their surface charge. Furthermore, the *ξ* measured in medium was about −8 mV, regardless of the nature of the IONPs’ surface and the media. Consequently, the proteins present in the medium tended to homogenize the surface charges among samples, hiding the inherent surface charge of uncoated and PLP-coated IONPs.

We also evaluated the optical properties of uncoated and PLP-coated IONPs by measuring their absorbance spectra by ultraviolet (UV)-visible spectroscopy (Figure S2). Since UV visible absorbance strongly depends on the environment surrounding IONPs, we measured the spectra in water (Figure S2a), as well as in protein-containing RPMI (Figure S2b) and DMEM (Figure S2c) media. Firstly, for uncoated IONPs, we found that the major peak around ~380 nm found in water shifted towards higher wavelengths when measured in RPMI and DMEM (Figure S2d). This has been previously reported for IONPs after their coverage by proteins [23], suggesting that proteins from the media bind to the surface of IONPs. Such peak shifts were also observed in both PLP-IONPsA and PLP-IONPsN (Figure S2e,f). Differences in absorbance intensities were also observed between the spectra of all samples measured in RPMI and DMEM as compared to water, confirming the presence of proteins in the environment [24].

### 2.3. Stability of PLP-Coated IONPs

The agglomeration state and the morphology of uncoated and PLP-coated IONPs were firstly studied by TEM. TEM micrographs of PLP-IONPsN and PLP-IONPsA in water (see Figure 1b,c, respectively) showed agglomerates with irregular shape for both coated IONP samples as compared to uncoated IONPs. Moreover, these agglomerates were larger in PLP-IONPsA (up to 1.5 μm) than in PLP-IONPsN (typically smaller than 500 nm). Such behaviour can be related to the probably low electrostatic repulsion interactions between the PLP-IONPsA caused by the relatively low negative charges (*ξ* was −3.9 ± 0.8 for PLP-IONPsA), which were not sufficient to provide colloidal stability.

In order to study the colloidal stability of IONPs in aqueous suspensions, *d_h_* of uncoated and PLP-coated IONPs were measured in water by DLS. The obtained values are given in Table 1. The *d_h_* of PLP-coated IONPs was larger than the uncoated IONPs, suggesting that IONPs agglomerated after the surface modification by coating, as observed in the TEM micrographs. However, some differences between TEM observations in dried samples and *d_h_* measured in water were expected due to the different state of the studied samples, i.e., dried powders and aqueous suspension. Furthermore, the *d_h_* of PLP-IONPsN was smaller than the *d_h_* of PLP-IONPsA, indicating that PLP-IONPsA were more agglomerated than PLP-IONPsN. This is consistent with the agglomerates observed by TEM (Figure 1b,c). Similar values of *d_h_* for all three types of IONPs were also found by two other size measurement methods, i.e., by the second DLS instrument and by centrifugal force (Figure S3).

It is known that NPs in biological environments are not only in aqueous medium, but are also in the presence of biomolecules, especially proteins. Importantly, the surface of NPs has been previously shown to be modified with proteins, forming the so-called protein corona [22]. In fact, the protein corona can change the aggregation state of NPs, which in turn alters their interaction with cells [25,26,27,28,29], their biodistribution [30,31] and their toxicity [32,33]. Therefore, in order to study the influence of the presence of proteins on the colloidal behaviour of IONPs, we measured *d_h_* in RPMI medium and DMEM (Table 1). First of all, *d_h_* measured in the presence of proteins in the two studied media were larger than *d_h_* measured in water, suggesting that the IONPs’ agglomeration was enhanced in biological medium in the presence of proteins. This was the case for uncoated and PLP-coated IONPs, confirming that proteins interact and bind to the surface of IONPs, modifying their aggregation state. In addition, PLP-IONPsN and PLP-IONPsA were agglomerated in both media and this behaviour was found to be more pronounced in both media than in water. This was expected due to the presence of the highly active chemical groups of PLP on the surface of PLP-coated IONPs, which can interact with biomolecules present in the environment, such as with proteins present in both media. However, for uncoated IONPs, the agglomeration was observed only in one medium, DMEM. In fact, the difference between RPMI and DMEM lies in their different salt and protein concentrations, which were shown to strongly influence the colloidal stability of NPs, their cytotoxicity and their cellular uptake [34]. These differences in salt and protein concentrations could therefore be the cause of the different stabilities of IONPs observed between the two media. In addition, the stability is not only medium-dependent, but also dependent on the coating type, i.e., the chemical nature of the surface upon coating, which drives the interaction with the bio-environment, which was also previously reported [34].

### 2.4. Toxicity Evaluation

**Figure 4:**
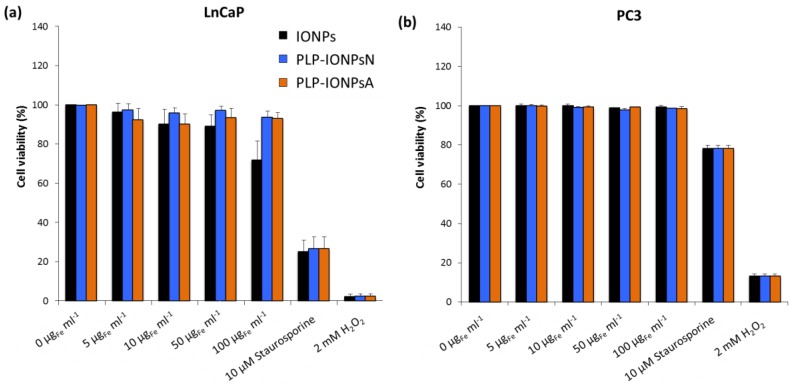
Viability of LnCaP (**a**) and PC3 (**b**) cells incubated for 24 h with different concentrations (0, 5, 10, 50 and 100 μg_Fe_ mL^−1^) of uncoated IONPs, PLP-IONPsN and PLP-IONPsA measured with the Annexin V/PI assay. The cell viabilities are percentages of viable cells treated with IONPs normalized with the number of viable cells without IONPs (0 μg_Fe_ mL^−1^). 10 μM of Staurosporine and 2 mM of H_2_O_2_ were used as positive controls.

The in vitro preliminary toxicity of different concentrations of uncoated and PLP-coated IONPs was measured on two human prostate cancer cell lines: (i) early metastatic cells from lymph nodes (LnCaP cells) and (ii) late metastatic cells from bones (PC3 cells). The toxicity of all samples was first assessed by the with the 3-(4,5-dimethylthiazol-2-yl)-5-(3-carboxymethoxypenyl)-2-(4-sulfophenyl)-2H-tetrazolium (MTS) test on LnCaP cells (Figure S4). All measured cell viabilities were above 75% (Figure S4), suggesting that uncoated and PLP-coated IONPs were not toxic for LnCaP cells up to a concentration of 100 μg_Fe_ mL^−1^ and that PLP is well tolerated by cells, as expected. However, due to the high variability of the cell viabilities measured with the MTS assay, no difference could be extracted between the studied samples. In fact, in absorbance-based toxicity tests, such as the MTS test, the absorbance of IONPs can modify the toxicity results, which can be corrected with our previously developed method [35]. Unfortunately, this method could not be applied in this study, because after few hours of incubation, 100% of the administrated IONPs were deposited and the IONPs fraction which stuck to the cells after the removal of the supernatant could not be estimated. Such issues with NP absorbance were not present in the fluorescence-based toxicity assays, such as the Annexin V and propidium iodide (PI) assays. We therefore also studied the toxicity of all samples on the LnCaP and PC3 cell lines by measuring the fluorescence of Annexin V and PI) by flow cytometry. In LnCaP cells, we found lower toxicities of uncoated and PLP-coated IONPs with the Annexin V/PI assay (Figure 4a) as compared to the MTS test. The Annexin V/PI assay performed on LnCaP cells showed that the PLP coating decreases the toxicity of IONPs (Figure 4a), while all viabilities of PC3 cells (Figure 4b), which are less sensitive to external stimuli and therefore more resistant than LnCaP cells, were ~100%, regardless the type of IONPs and the IONP concentration. Therefore, by decreasing the toxicity of IONPs in vitro, PLP, which is involved in essential in vivo biological functions and is known to be biocompatible, is a promising molecule for its use in biomedical applications.

### 2.5. Interaction of IONPs with Cells

In order to study the potential use of PLP simultaneously as a molecule for targeting tumour cells and to exploit the role of PLP in essential body functions such as metabolism, we investigated the influence of PLP on the cellular uptake of IONPs. Firstly, we studied the uptake of PLP-IONPsN and PLP-IONPsA at different concentrations in LnCaP and PC3 cells by extracting additional information from the above-presented flow cytometry results. We measured the forward scattered (FSC) light, which is proportional to the cell surface area and therefore their size, and the side scattered (SSC) light, which is a measure of the cell granularity and intracellular structure complexity. In fact, it was recently shown that SSC can be used to measure the amount of NPs taken up by cells, because NP uptake modifies the intracellular structure and increases cell granularity [36]. The SSC in function of the FSC for LnCaP and PC3 cells is shown in Figures S5 and S6, respectively, where each point represents a cell. We can observe an increase of points/cells with higher SSC values with an increase in IONP concentration (for instance the first vs. the last row in Figures S5 or S6), meaning that these cells have higher granularity. If we plot the sum of the number of cells (cell counts) found for each SSC value in Figures S5 and S6, we obtained the result showed in Figure S7. Indeed, the broadening of the SSC-count’s peak and the shift of the peak towards higher values for higher IONP concentrations in both LnCaP and PC3 cells were observed for both uncoated and PLP-coated IONPs. Furthermore, the largest shift in the SSC towards higher values, and therefore the highest cell granularity, was observed for PLP-coated IONPs in PC3 cells. Therefore, the uptake of PLP-coated IONPs in PC3 cells was higher than that of uncoated IONPs.

**Figure 5:**
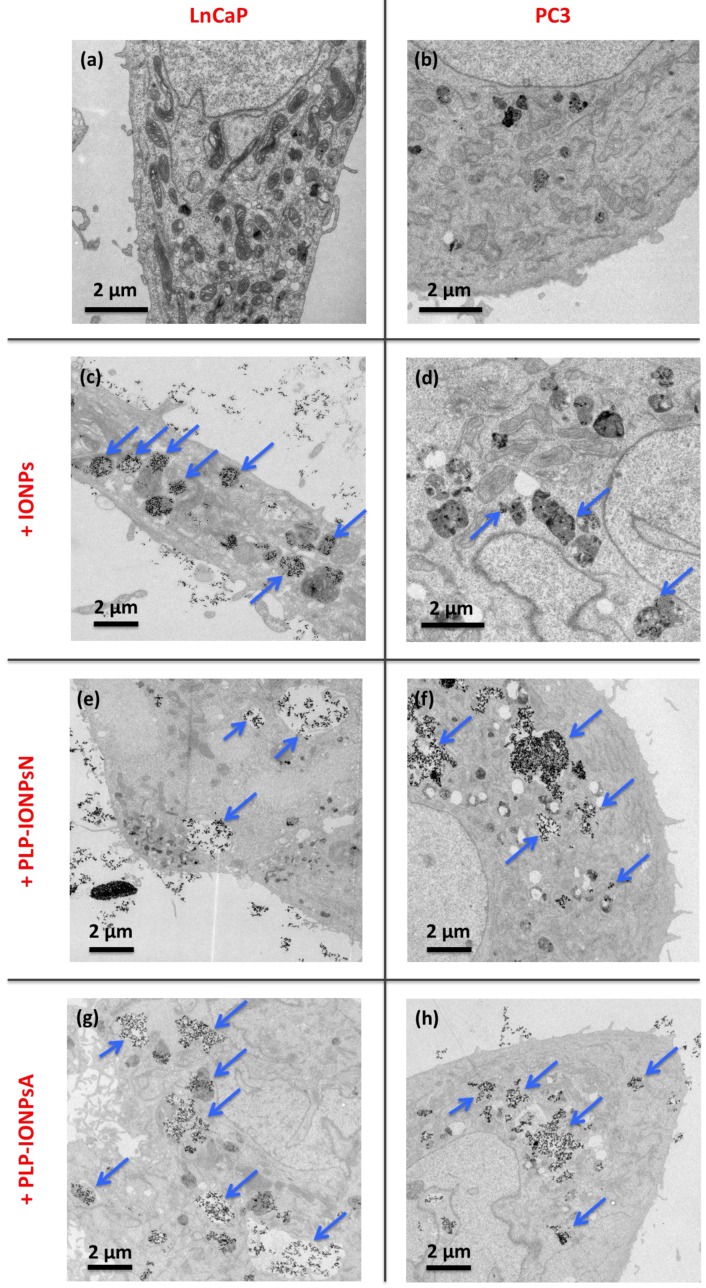
Representative TEM micrographs of 50 nm-thick sections of LnCaP (**a**,**c**,**e**,**g**) and PC3 (**b**,**d**,**f**,**h**) cells without IONPs (**a**,**b**) or incubated for 24 h with 100 μg_Fe_ mL^−1^ uncoated IONPs (**c**,**d**), PLP-IONPsN (**e**,**f**) and PLP-IONPsA (**g**,**h**).

In order to study the differences seen in the uptake in more details, we did a TEM study, which allowed us to locate IONPs inside cells and to observe the morphologies of cells and vesicles. For this purpose, we prepared 50-nm thick sections of cells, and stained it with uranyl acetate to enhance the cellular contrast, which was examined by TEM. Representative TEM micrographs of these 50 nm-thick sections of LnCaP and PC3 cells incubated with 100 μg_Fe_ mL^−1^ of uncoated and PLP-coated IONPs are shown in Figure 5 and Figure S8. Firstly, in LnCaP cells, the amount of IONPs taken up was similar between uncoated and PLP-coated IONPs, as well as between PLP-IONPsN and PLP-IONPsA. However, the vesicles containing IONPs were different between uncoated IONPs and PLP-coated IONPs. For uncoated IONPs, these vesicles were found already deep in the intracellular space, while the vesicles containing PLP-IONPsN and PLP-IONPsA were in both cases located rather at the edges of the cells. Therefore, the uptake process seemed to be more advanced and therefore faster for uncoated IONPs as compared to PLP-coated IONPs. In addition, uncoated IONPs were mostly found in small structured vesicles (~1 μm in diameter), but PLP-coated IONPs were also found in empty vesicles with much larger diameters. The morphology of the vesicles was also the major difference found between PLP-IONPsN and PLP-IONPsA; while vesicles containing PLP-IONPsN were smaller than 1 μm in diameter and mostly structured (see arrows in Figure 5e), they were much larger (>1–5 μm), as well as unstructured and empty for PLP-IONPsA (see arrows in Figure 5g). In LnCaP cells, we therefore essentially found differences in the morphological properties of vesicles containing IONPs, rather than in the amount of IONPs taken up, which could be due to a difference in the uptake mechanism involved between uncoated and PLP-coated IONPs, but also between PLP-IONPsN and PLP-IONPsA.

In PC3 cells, the amount of uncoated IONPs found inside cells was much smaller than in LnCaP cells, as also seen above with the SSC shift in the flow cytometry results. In addition, IONPs were hardly distinguishable from the intracellular dark structures (see arrows in Figure 5d). Interestingly, the amount of PLP-IONPsN and PLP-IONPsA found inside PC3 cells was much larger than that of uncoated IONPs, that is also in agreement with the above given results from the flow cytometry. PLP therefore increases the amount of IONPs taken up in PC3 cells, which was not the case in LnCaP cells, suggesting that PLP modifies the interaction of IONPs specifically with PC3 cells. Furthermore, the uptake of PLP-IONPsN was similar to that of PLP-IONPsA, both being more advanced than in LnCaP cells. IONPs were very rarely found close to the cellular surface, but rather deep inside the cells and even around the nucleus. In addition, the morphology of the vesicles containing PLP-coated IONPs was similar between PLP-IONPsN and PLP-IONPsA; in both coated samples, IONPs were located in large vesicles with diameters of ~2–4 μm (see arrows in Figure 5f,h). However, a large difference in the vesicle morphologies was found between uncoated and PLP-coated IONPs. Consequently, in PC3 cells, the uptake seems to be similar between PLP-IONPsN and PLP-IONPsA, but different from that of uncoated IONPs. The difference mainly lay in the amount of IONPs found inside cells, even though differences in the morphology of vesicles were also observed between uncoated and PLP-coated IONPs. This was not the case in LnCaP cells, in which the intracellular amounts of uncoated and PLP-coated IONPs were similar, but the vesicle morphologies were different. Consequently, we show that the uptake is cell specific, as previously reported for folic acid coated IONPs [37], and that PLP modulates the uptake of IONPs in both LnCaP and PC3 cells. More precisely, PLP modified the uptake of IONPs from a mechanistic point of view in the first cell line, but both in a mechanistic manner and by changing the amount of IONPs taken up in the second one.

The differences observed in the amounts of all studied samples taken up by cells and the morphology of the vesicles containing these IONPs could be due to a difference in IONPs and/or in the uptake mechanism. In fact, the major difference between LnCaP and PC3 cells resides in the different expression of the prostate specific membrane antigen (PSMA) [38,39]. While PC3 cells do not express PSMA, LnCaP cells do. Interestingly, small molecules (e.g., S,S-2-(3-(5-amino-1-carboxypentyl)-ureido)-pentanedioic acid, ACUPA) have been used to direct NPs to PSMA-positive prostate cancer cells [40,41]. However, the chemical structures of these molecules are very different to that of PLP, suggesting that the differences observed in the uptake of PLP-IONPs between LnCaP and PC3 cells are not mediated by the presence and the absence of PSMA, respectively. Therefore, the observed differences in the uptake between the studied samples could rather be caused by the samples’ physico-chemical properties, such as the different agglomeration states and surface charges described above between the two PLP-coated IONPs, but also between uncoated and PLP-coated IONPs, which could be at the origin of these different uptake mechanisms. It is known that PLP serves as a coenzyme for numerous enzymatic reactions (e.g., amino acid biosynthesis and catabolism via their transamination, gluconeogenesis, hemoglobin synthesis, etc.), [10] which occur in the intracellular space. PLP was shown to be transported in the blood by being attached to albumin in order to reach the tissues where these enzymatic reactions occur [42]. However, while the non-phosphorylated form pyridoxal is known to cross the cellular membrane [43,44,45], only very little is known about the cellular uptake of the membrane impermeable PLP [46]. It seems that the cellular uptake of PLP is carrier-mediated [44], but deep knowledge of the transporters involved in these mechanisms is lacking [43]. Interestingly, one of the rear studies, which investigated the uptake of PLP in cells, showed that PLP is rapidly taken up by human red blood cells [47]. Here we show for the first time that PLP-coated NPs are taken up by tumour cells in a different manner than uncoated IONPs. While IONPs are known to be taken up via the endocytosis-mediated pathway [48,49], the uptake of PLP-coated IONPs could be mediated via the poorly-investigated carrier that is involved in the uptake of the membrane-impermeable PLP. Furthermore, we show that the orientation of PLP at the IONPs’ surface modifies the uptake of IONPs, which was especially found in PC3 cells, which suggests that the uptake not only depends on the chemical nature of the coating, but also on the orientation of this molecule on the surface of IONPs. Our results are a step forward in the understanding of the cellular uptake of PLP bound to NPs and show that PLP controls the interaction of IONPs with cells, as previously reported for other types of coatings [34,50,51,52,53,54]. This could be used to modulate the cellular uptake of NPs and further studies should investigate how PLP at the IONPs’ surface affects other biological effects, such as the NPs’ biodistribution, blood circulation and metabolism.

### 2.6. Potential as an MRI Contrast Agent

**Figure 6:**
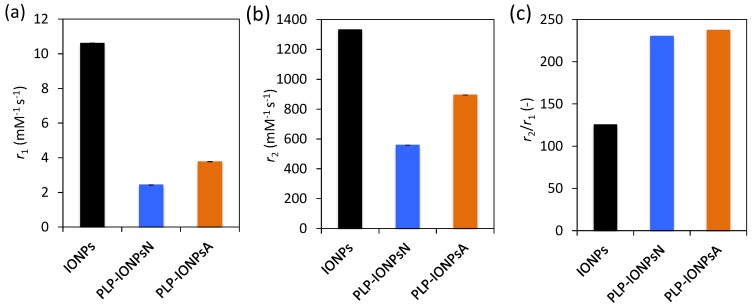
Magnetic resonance imaging (MRI) longitudinal *r*_1_ (**a**) and transverse *r*_2_ relaxivity (**b**) and the relaxivity ratio, *r*_2_/*r*_1_ (**c**) measured at room temperature at 3 T for uncoated IONPs, PLP-IONPsN and PLP-IONPsA.

IONPs are known to be very promising as contrast agents for MRI, because they shorten the relaxation time of neighbouring protons, especially the transverse relaxation time T_2_, therefore they are considered as T2 contrast agents. In order to evaluate the potential use of PLP-coated IONPs for diagnostics by MRI, we measured their longitudinal (*r*_1_) and transverse (*r*_2_) relaxivities. Figure 6a,b and c show the *r*_1_ and *r*_2_ values, as well as the relaxivity ratio *r*_2_/*r*_1_ for uncoated IONPs, PLP-IONPsN and PLP-IONPsA measured at room temperature in a clinical 3 T MRI scanner. Firstly, we measured lower *r*_1_ values for PLP-coated IONPs, as compared to uncoated IONPs. Previous studies have reported that the addition of a coating molecule, i.e., an increase of the size, reduces the *r*_1_ relaxivity [55]. The most important parameter for IONPs, which are classified as T2 contrast agents, is the *r*_2_ relaxivity. This parameter reflects the shortening of the relaxation rate (1/T_2_) as function of a IONPs concentration, meaning that higher concentrations of IONPs accelerate the spin–spin relaxation time of adjacent water molecules, resulting in the faster decay of the MRI signal. We measured a smaller *r*_2_ relaxivity for PLP-coated IONPs as compared to uncoated IONPs, which was previously observed after the addition of a coating, i.e., an increase of the size upon coating, which could make the T_2_ shortening capabilities less efficient [55,56,57]. Furthermore, we have found above agglomerates in both PLP-IONPsN and PLP-IONPsA, which were previously shown to affect the *r*_2_ relaxivity [58]. However, the obtained *r*_2_ values of 559 mM^−1^s^−1^ and 894 mM^−1^s^−1^ for PLP-IONPsN and PLP-IONPsA, respectively, were unexpectedly high for coated IONPs. This could be explained by the relatively thin PLP coating. Indeed, the obtained *r*_2_ values for PLP-IONPsN and PLP-IONPsA are still much higher than in most of the reported IONPs coated with larger molecules (e.g., polyethylene glycol, dextran), which had at 3 T *r*_2_ values between 50–164 mM^−1^s^−1^ [59,60,61]. Thus, our results confirm the importance of the coating thickness for the *r*_2_ relaxivity of IONPs.

Besides the high *r*_2_ value, the *r*_2_/*r*_1_ ratio was two times larger for PLP-coated IONPs than uncoated IONPs. It has been previously shown that decreasing the coating thickness increases the *r*_2_*/r*_1_ ratio [55]. In fact, contrast agents with both large *r*_2_ and *r*_2_*/r*_1_ ratios are necessary for their use as efficient T2 contrast agents. Consequently, given the high *r*_2_ and *r*_2_*/r*_1_ ratio that we measured for PLP-coated IONPs, the use of PLP as a small coating and at the same time targeting molecule is highly beneficial for their superior properties for T_2_-weighted imaging as compared to products found in the literature.

## 3. Materials and Methods

### 3.1. Synthesis of IONPs

IONPs were synthesized following a protocol modified from Bonvin *et al*. and described previously [15]. Briefly, IONPs were synthesized by co-precipitation in combination with a hydrothermal treatment performed at 120 °C for 15 h. At the end of the synthesis, the obtained IONPs were kept in 10 mM HNO_3_ at 4 °C.

### 3.2. Coating of IONPs with PLP

Two types of coated IONPs were prepared: 

(1) PLP-IONPsN: An IONP suspension (in 10 mM HNO_3_), which contained 50 mg of IONPs (35 mg Fe) was mixed with water to result in 35 mL of suspension. The pH of the IONP suspension was adjusted to 7 with ~700 μL of 0.25% ammonia solution. The IONPs were separated on a strong magnet and the supernatant was removed; 15 mL of water was added and the IONP suspension was sonicated for 6 min to remove IONPs agglomerates. The pH of the resulting IONP suspension was 7.0.

(2) PLP-IONPsA: An IONP suspension (in 10 mM HNO_3_), which contained 50 mg of IONPs (35 mg Fe) was mixed with water to result in 10 mL of suspension. The pH of the resulting IONP suspension was 2.5.

For both PLP-IONPsN and PLP-IONPsA, 68 mg of pyridoxal phosphate (FluoroChem, 98% purity) were dissolved in 15 mL water. 5 mL of the dissolved pyridoxal phosphate were added to the IONPs suspensions, which were rotated for 5 min with a rotator (tube rotator from VWR International) placed on a shaker at 500 rpm. This procedure was repeated until the whole solution of pyridoxal phosphate was added to the IONP suspension. The volume was adjusted to 35 mL with water to approximately reach a final concentration of 1 mg_Fe_ mL^−1^ and the IONP suspensions were rotated for 30 min with the rotator placed on the shaker at 500 rpm. After 30 min, the IONP suspensions were dialyzed (Spectra/Por^®^; 12–14 kDa) against water for 72 h by changing the dialysis solution every 10–12 h, and finally, the obtained suspensions were stored at 4 °C.

### 3.3. Characterization of PLP-Coated IONPs

For transmission electron microscopy (TEM), 5 μL drops of IONP suspensions were deposited on holey carbon grids (Plano GmbH, Wetzlar, Germany) placed onto absorbing filter paper and left to dry at room temperature. TEM micrographs were taken with a Talos F200X FEI electron microscope (FEI, Gräfelfing, Germany) operated at an acceleration voltage of 200 kV and coupled to a CMOS-based FEI CETA 4000 × 4000 camera (FEI, Gräfelfing, Germany). The primary particle size of uncoated IONPs (diameter of IONPs, in the text referred as *TEM diameter*) of 1000 IONPs was measured manually from randomly taken TEM micrographs using ImageJ software (the National Institutes of Health, Bethesda, MD, USA).

For all samples the iron concentration was determined by ICP-EOS with ICP-EOS 9000 (Shimadzu, Duisburg, Germany). For this purpose, a volume of 80 μL of IONPs stock suspension of as-synthesized-IONPs was mixed with 920 μL of 6 M HCl. After three days, the IONPs were fully dissolved and 500 μL of the obtained solution containing the dissolved Fe-ions was diluted in 2.5 mL of water.

The hydrodynamic diameters (*d_h_*) and the zeta potentials of 1 mL IONP suspension at a concentration of 100 μg_Fe_ mL^−1^ in water, RPMI medium and Dulbecco’s Modified Eagle Medium (DMEM) were measured at room temperature in acrylic cuvettes (Sarstedt, Nümbrecht, Germany) with a Zetasizer Nano ZS (Malvern Instruments, Worcestershire, UK). The reported values of *d_h_* were obtained from the average of 3 × 12 measurements. The refractive index of γ-Fe_2_O_3_ and absorbance were set to 2.95 and 0.1, respectively. The *d_h_* of IONP suspension in water were also measured by laser diffraction with the Mastersizer 3000 (Malvern Instruments, Worcestershire, UK) using the small volume sample dispersion unit. The refractive index and density of γ-Fe_2_O_3_-IONPs were set to 3.01 and 4.92 g mL^−1^, respectively, while the absorbance was set to 0.1. Additionally, the *d_h_* of 50 μL of IONP suspensions at 200 μg_Fe_ mL^−1^ were measured at room temperature by centrifugal force with a disc centrifuge (CPS Instruments, Oosterhout, Neatherland) at 22,000 rpm. The refractive index and density of γ-Fe_2_O_3_-IONPs were set to 2.95 and 4.92 g mL^−1^, respectively, while the absorbance was set to 0.1. A sucrose gradient from 8% up to 24% sucrose was used. 

UV-visible spectra of IONP suspensions in water, RPMI medium and DMEM were measured at concentrations of 100 μg_Fe_ mL^−1^ in Brand^®^ UV-cuvettes with a Cary 100 Bio spectrometer (Agilent Technologies, Waldbronn, Germany) between 190 and 900 nm. The average time was set to 0.1 s, the data interval to 1 nm and the scan rate to 600 nm min^−1^.

IONP suspensions were lyophilized for four days with an alpha 1–2 Laboratory dryer (LD) plus freeze dryer. Fourier transform IR (FTIR) spectra of IONPs powder pellets were obtained with the Spectrum One spectrometer (series: 69288, Perkin Elmer, Schwerzenbach, Switzerland). Transmittance from 4600 cm^−1^ to 400 cm^−1^ were given as the average of measured 64 scans for each curve with a resolution of 4.00 cm^−1^.

X-ray Photoelectron Spectroscopy (XPS) measurements were carried out using a PHI VersaProbe II scanning XPS microprobe (Physical Instruments AG, Meylan, France). Analysis was performed using a monochromatic Al Kα X-ray source of 24.8 W power with a beam size of 100 μm. The spherical capacitor analyser was set at a 45° take-off angle with respect to the sample surface. The pass energy was 46.95 eV yielding a full width at the half maximum of 0.91 eV for the Ag 3d 5/2 peak. Curve fitting was performed using the PHI Multipak software (Blue Scientific, Cambridge, UK).

### 3.4. Binding Strength of PLP Chemical Groups on IONPs

In order to evaluate the binding strength of both the deprotonated and protonated phosphoryl and phenolic groups to the surface of an IONP, the Quantum opEn-Source Package for Research in Electronic Structure, Simulation, and Optimization (ESPRESSO) [62] package was used to perform DFT calculations of a PLP molecule, which carries these groups, adsorbed to a (110) surface of magnetite (Fe_3_O_4_). Despite the different iron-oxide phase, the magnetite surface can serve as a model for the more complex maghemite surface, because the chosen surface is Fe^3+^ terminated [63] and has the same ion arrangement, however without a random arrangement of oxygen vacancies. Thus, this surface should be chemically similar to the ones exposed by a typical IONP. Orientations of the molecule were chosen so that always only one of the functional groups interacted with the surface. Using the same calculation setup as in Aschauer and Selloni [63], structures were relaxed until forces converged below 0.05 eV Å^−1^ and the adsorption energy was computed as: ∆*E*_ads_ = − [*E*_surf+molec_ −(*E*_surf_ + *E*_molec_)], where *E*_surf+molec_ is the total energy of the surface with adsorbed molecule, while *E*_surf_ and *E*_molec_ are the total energies of the isolated surface and molecule, respectively. Within this convention, a positive adsorption energy indicates favourable binding to the surface. Figure 2 shows the final relaxed configurations for the both chemical groups in the deprotonated and protonated states.

### 3.5. Cellular Uptake Study by TEM

LnCaP and PC3 cells were cultured in RPMI 1640 medium (American Type Culture Collection, ATCC, Wesel, Germany) and DMEM GlutaMAX with 25 mM high glucose and 1 mM sodium pyruvate (Gibco, Thermo Fisher Scientific, Darmstadt, Germany), both supplemented with 10% fetal bovine serum (Life Technologies, Thermo Fisher Scientific, Darmstadt, Germany) and 1.5% 10,000 U mL^−1^ Penicillin Streptomycin (Life Technologies, Thermo Fisher Scientific, Darmstadt, Germany). 40,000 LnCaP cells and 50,000 PC3 cells per well were cultured on plastic 13 mm diameter sterile coverslips (Nunc^TM^ Thermanox^TM^) in 12 well plates at 37 °C. Cells were exposed to 1 mL of 100 μg_Fe_ mL^−1^ of IONPs for 24 h. Cells treated only with medium served as negative controls. After 24 h, cells were washed three times with PBS and fixed for 1 h at room temperature with 2% paraformaldehyde and 2.5% glutaraldehyde in PBS (pH 7.4). After 1 h, coverslips were washed three times with 0.1 M cacodylate buffer (pH 7.4), stained with 1% osmium tetroxide in 0.1 M cacodylate buffer at room temperature for 1 h and with 2% uranyl acetate in water for 40 min. After dehydrating the coverslips with an increasing percentage of ethanol, up to 100%, they were then embedded in 50% durcupan (in ethanol) for 30 min and in 100% durcupan for 2 h. The resin was left to polymerize overnight at 60 °C. The next day, the resin embedded samples were separated from the glass slides by plunging them alternately into liquid nitrogen and hot water. The cells were then thin sectioned at a thickness of 50 nm with a diamond knife (Diatome, Leica Mikrosysteme Vertrieb GmbH, Wetzlar, Germany) and ultramicrotome (Leica Mikrosysteme Vertrieb GmbH, Wetzlar, Germany) and collected on a formvar film on copper slot grids. Samples were imaged with a transmission electron microscope operating at 80 kV (FEI, Gräfelfing, Germany, Tecnai Spirit, FEI, Gräfelfing, Germany).

### 3.6. In Vitro Toxicity Study by the MTS Assay

40,000 LnCaP cells per well were cultured in 96-well plates at 37 °C, and exposed to 100 μL of different concentrations of IONPs (0, 1, 5, 10, 50 and 100 μg_Fe_ mL^−1^) for 24 h. Cells treated only with medium served as negative controls. After 24 h incubation, the supernatant of each well were removed. 100 μL of MTS solution (CellTiter 96^®^ Aqueous One Solution Cell Proliferation Assay from Promega, diluted six times in medium) was added to the cells. After 2 h incubation, the absorbance of the formazan product was measured with a microplate reader (Tecan Infinite M200, Tecan, Männedorf, Switzerland) at a wavelength of 490 nm. The cell viabilities were calculated as the absorbance in cells treated with IONPs normalized with the absorbance of cells without IONPs (0 μg_Fe_ mL^−1^).

### 3.7. Cellular Uptake and Annexin V/PI In Vitro Toxicity Study by Flow Cytometry

350,000 LnCaP cells and 200,000 PC3 cells per well were cultured in six well plates, and exposed to 2.5 mL of different IONP concentrations (0, 5, 10, 50 and 100 μg_Fe_ mL^−1^) for 24 h. Cells treated only with medium served as negative controls, and cells treated with 10 μM of staurosporine (diluted from 1 mM solution in DMSO, Sigma-Aldrich, Taufkirchen, Germany) for 20 h and 2 mM H_2_O_2_ (diluted from 3% stock solution, Fluka, Sigma-Aldrich, Taufkirchen, Germany) for 4 h were used as positive controls. After 24 h incubation, cells were washed once with PBS and detached from the wells with 0.05% trypsin-EDTA (Gibco). Cells were centrifuged and resuspended in 100 μL of Annexin V Binding Buffer (BioLegend, London, UK). 12.5 μL of 1/25 APC-Annexin V solution (BioLegend, London, UK) were added to the cells, which were incubated for 25 min at room temperature in the dark. 5 μL of PI and 100 μL of Annexin V Binding Buffer were then added. Samples were analyzed by flow cytometry (Accuri C6, BD Biosciences, Allschwil, Switzerland). APC-Annexin V was detected with λ_exc_ = 640 nm and λ_em_ = 675 ± 12.5 nm and PI was detected with λ_exc_ = 488 nm and λ_em_ = 585 ± 20 nm. The side scattered (SSC) light was measured in function of the forward scattered (FSC) light, to determine the cell granularity and consequently the cellular uptake of IONPs [36]. The cell viabilities are reported as cells negative for both Annexin V and PI. For both the MTS test and the Annexin V/PI assay, all experiments were performed in triplicate. Results are given as means (with standard deviations) of the values obtained in these triplicates.

### 3.8. MRI

The IONPs were suspended in 2% agarose gel in 0.5 mL Eppendorf cups to obtain final concentrations ranging from 0 to 30 µg_Fe_ mL^−1^. All cups were placed in a water-containing phantom for subsequent *T*_1_ and *T*_2_ measurements, which were performed at room temperature on a 3.0 T clinical MRI scanner (MAGNETOM Prisma, Siemens AG, Erlangen, Germany), and data was acquired using a body array coil. The measurement of the longitudinal relaxation times *T*_1_ was performed using a 2 dimensional spin echo sequence preceded by a 180° inversion pulse with different inversion times (TI). Measurements of the transversal relaxation times *T*_2_ were performed with a 2D spin echo sequence with variation of the echo time (TE). Imaging parameters for longitudinal relaxation times *T*_1_ were as follows: TE 6.3 ms, slice thickness 5 mm, Field of View (FOV) 250 × 150 mm^2^, matrix 384 × 310, radiofrequency (RF) excitation angle 90°, receiver bandwidth of 651 Hz/pixel, TI 23, 50, 75, 100, 125, 150, 175, 200, 300, 400, 500, 1000, 5000, 9000 ms. Imaging parameters for transversal relaxation times *T*_2_: TE 6.3, 8, 10, 15, 20, 25, 30, 35, 65, 250 ms, slice thickness 5 mm, FOV 250 × 150 mm^2^, matrix 384 × 310, RF excitation angle 90°, receiver bandwidth of 651 Hz/pixel. The magnetization was allowed to recover for 15 s between acquisitions. The signal evolution S as function of TI and TE was fitted to derive the *T*_1_ and *T*_2_ of each γ-Fe_2_O_3_ NPs suspension respectively, and is described as follows:

(1) $$S\left(TE\right)=S\left(0\right)e^{-\frac{TE}{T_{2}}}+C$$

(2) $$S\left(TI\right)=S\left(0\right)\left(1-2e^{-\frac{TI}{T_{1}}}\right)$$

The *T*_1_ and *T*_2_ values as function of their γ-Fe_2_O_3_ NPs concentration were subsequently fitted to obtain the relaxivities *r*_1_ and *r*_2_ described as:

(3) $$\frac{1}{T_{1,2}}=\frac{1}{T_{1,2}\left[0\right]}+r_{1,2}\left[\gamma -Fe_{2}O_{3}\right]$$

All data were analyzed and fitted with Matlab (The MathWorks, Natick, MA, USA).

## 4. Conclusions

In this work we investigated for the first time the potential of PLP to be simultaneously used as a coating molecule and targeting molecule via the direct attachment of PLP to IONPs. By controlling the pH of the coating reaction, PLP was oriented differently at the IONPs’ surface, which resulted in different physico-chemical properties of PLP-coated IONPs, such as surface charges, zeta potential, hydrodynamic radius and agglomeration state. In addition, we didn’t observe any toxicity on both LnCaP and PC3 cells, giving PLP interesting biocompatibility features for the coating of NPs for medical applications. Furthermore, we measured high *r*_2_ values for the use of PLP-coated IONPs as contrast agents for MRI. Importantly, we found that PLP controlled the interaction of IONPs with both studied cell lines, LnCaP and PC3. In LnCaP cells, PLP modified the morphology and diameter of the IONP-containing vesicles, while in PC3 cells the amount of IONPs taken up was different. Therefore, the results of this study provide evidence that PLP can be used as a coating molecule and targeting agent around IONPs, allowing us to tune the interaction of IONPs with cells. While the cellular uptake of PLP has been so far poorly studied, we provide the first understanding of the effect of PLP bound to IONPs in this process. Further studies on the biodistribution, blood circulation or metabolism of PLP-coated IONPs could provide more insights into the potential use of PLP as a coating and targeting molecule for various medical applications.

## Figures and Tables

**Table 1:** Hydrodynamic diameter (*d_h_*) and zeta potential (*ξ*) measured by DLS of 100 μg_Fe_ mL^−1^ uncoated IONPs, PLP-IONPsN and PLP-IONPsA measured in water (pH ~7.0), in Roswell Park Memorial Institute (RPMI) medium and in Dulbecco’s Modified Eagle Medium (DMEM) (pH ~7.6).

	*d_h_* in Water (nm)	*d_h_* in RPMI (nm)	*d_h_* in DMEM (nm)	*ξ* in Water (mV)	*ξ* in RPMI (mV)	*ξ* in DMEM (mV)
IONPs	23 ± 8	114 ± 39	1352 ± 562	5.0 ± 2.0	−7.6 ± 0.6	−9.2 ± 0.7
PLP-IONPsN	1065 ± 296	1893 ± 440	1657 ± 552	−16.7 ± 0.6	−7.6 ± 0.7	−7.5 ± 0.6
PLP-IONPsA	1592 ± 537	1752 ± 379	1685 ± 1074	−3.9 ± 0.8	−7.9 ± 0.8	−8.0 ± 0.9

## Supplementary Materials

The following are available online at www.mdpi.com/2079-4991/7/8/202/s1, Figure S1: Overall XPS spectra and their Fe 2p, N 1s, C 1s and P 2p peaks, Table S1: Atomic percentages of Fe, 0, C and N obtained by XPS, Figure S2: Absorbance measured by UV-visible spectroscopy in water, in RPMI medium and in DMEM, Figure S3: Number weighted distribution of the hydrodynamic diameter of IONPs measured by dynamic light scattering with two instruments and by centrifugal force, Figure S4: Forward scatter vs. side scatter plot of LnCaP cells, Figure S5: Forward scatter vs. side scatter plot of PC3 cells, Counts vs. area of the side scatter plot of LnCaP and PC3 cells, Figure S7: Representative TEM micrographs of 50 nm-thick cross-sections of LnCaP and PC3 cells.
