# Supplementary material for: Versatility of Pyridoxal Phosphate as a Coating of Iron Oxide Nanoparticles
Source: Nanomaterials (Basel). 2017 Jul 29;7(8):202. doi: 10.3390/nano7080202 (PMC5575684; doi:10.3390/nano7080202)
Supplement: Supplementary file 1 [file nanomaterials-07-00202-s001.pdf]

Supplementary data

Versatility of pyridoxal phosphate as a coating of iron oxide nanoparticles

Debora Bonvin,[1] Ulrich Aschauer,[2] Jessica A.M. Bastiaansen,[3,4] Matthias Stuber,[3,4] Heinrich Hofmann,[1] and Marijana Mionić Ebersold[1,3,4*]

[1]Powder Technology Laboratory, Insitute of Materials, Ecole polytechnique fédérale de Lausanne, Switzerland.

[2] Department of Chemistry and Biochemistry, University of Bern, Bern, Switzerland.

[3]Department of Radiology, University Hospital (CHUV) and University of Lausanne (UNIL), Switzerland.

[4]Center of Biomedical Imaging (CIBM), Lausanne, Switzerland.

*Corresponding author E-mail addresses: marijanamionic@gmail.com; Marijana.Mionic-Ebersold@chuv.ch.

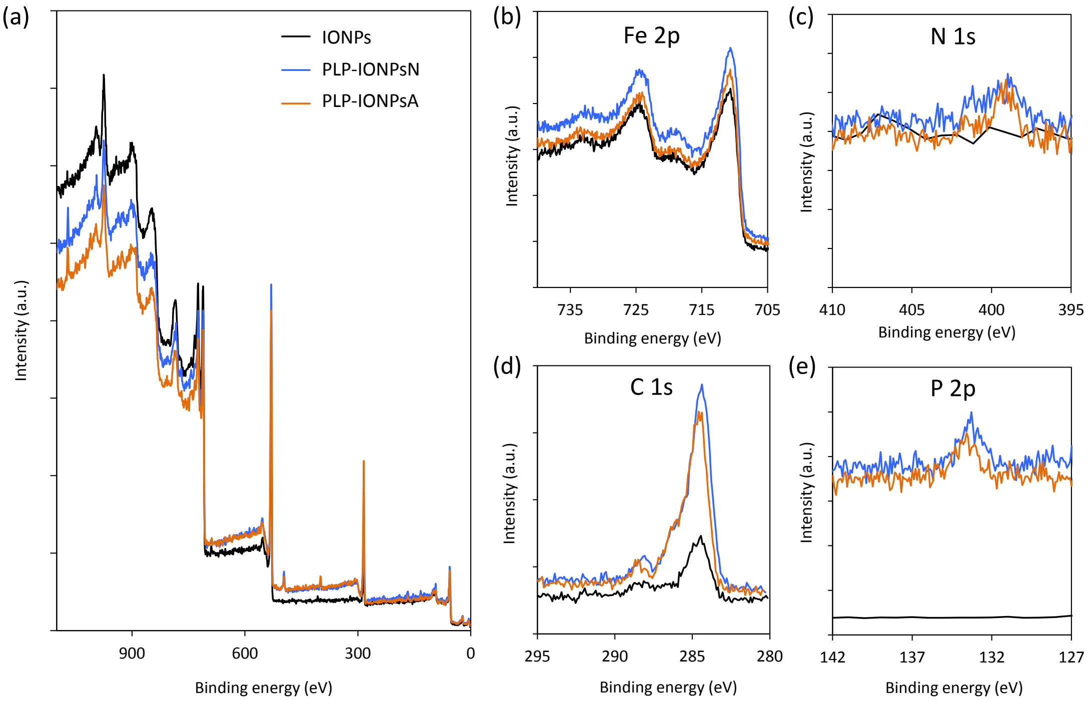

Fig. S1: Overall XPS spectra of uncoated IONPs, PLP-IONPsN and PLP-IONPsA (a), as well as their Fe 2p (b), N 1s (c), C 1s (d) and P 2p (e) peaks.

Table S1: Atomic percentages of Fe, O, C and N obtained by XPS in uncoated IONPs, PLP-IONPsN and PLP-IONPsA.

Atomic %	**Fe 2p**	**O 1s**	**C 1s**	**N 1s**	**P 2p**
IONPs	32.0	50.6	17.3	-	-
PLP-IONPsN	21.9	43.0	32.0	2.1	1.1
PLP-IONPsA	18.6	44.3	33.6	2.2	1.3

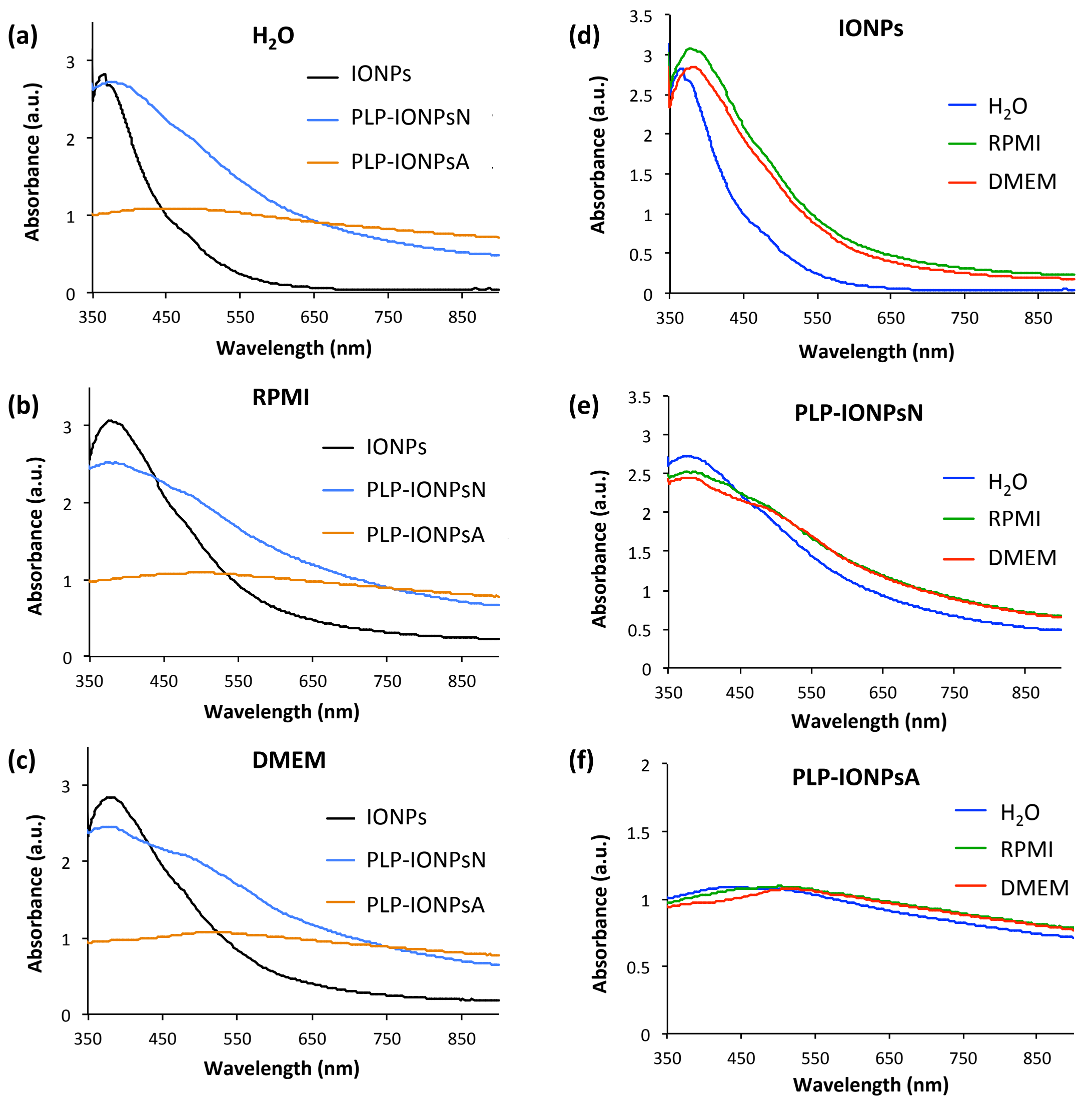

Fig. S2: Absorbance measured by UV-visible spectroscopy in H_2O (a), in RPMI medium (b) and in DMEM (c) of uncoated IONPs (d), PLP-IONPsN (e) and PLP-IONPsA (f).

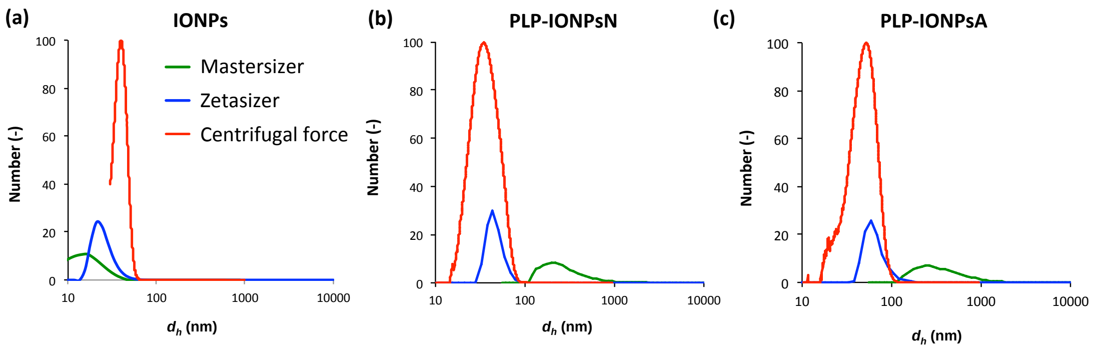

Fig. S3: Number weighted distribution of the hydrodynamic diameter, d_h, of uncoated IONPs (a), PLP-IONPsN (b), and PLP-IONPsA (c) measured by dynamic light scattering with the two instruments (Mastersizer or Zetasizer), or by centrifugal force.

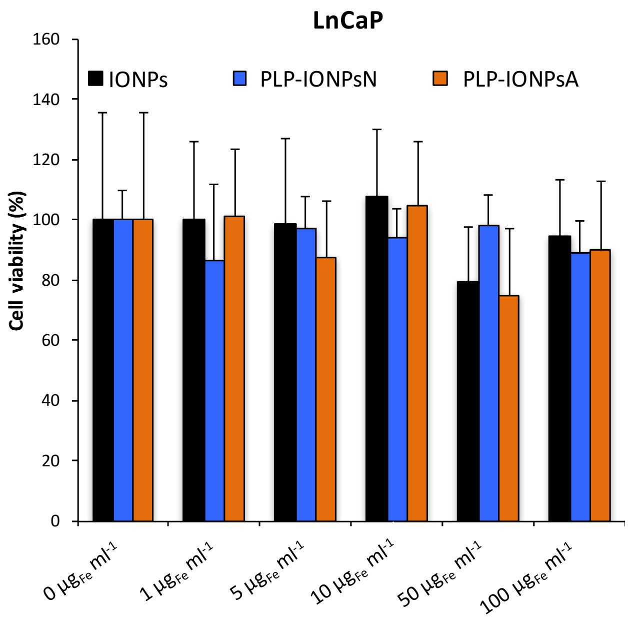

Fig. S4: Viability of LnCaP cells incubated for 24 h with different concentrations (0, 5, 10, 50 and 100 μg_{Fe} ml^{-1}) of uncoated IONPs, PLP-IONPsN and PLP-IONPsA measured with the MTS assay. The cell viabilities are percentages of viable cells treated with IONPs normalized with the number of viable cells without IONPs (0 μg_{Fe} ml^{-1}).

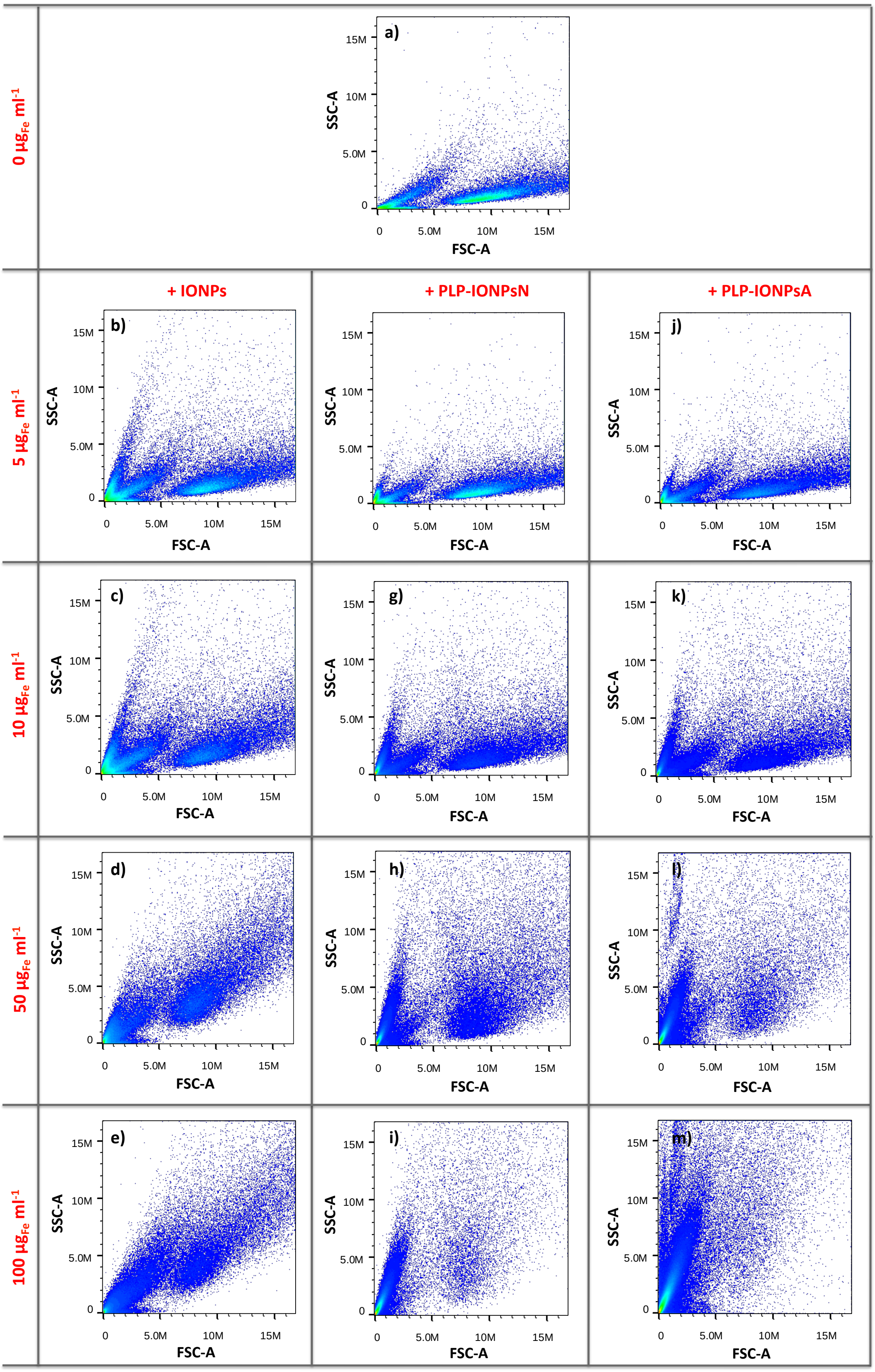

Fig. S5: Forward scatter (FSC) *vs* side scatter (SSC) plot of LnCaP cells without IONPs (0 μg$_{Fe}$ ml^{-1}; a) or treated for 24 h with different concentrations (5, 10, 50 and 100 μg$_{Fe}$ ml^{-1}) of uncoated IONPs (b, c, d, e), PLP-IONPsN (f, g, h, i) or PLP-IONPsA (j, k, l, m). SSC-A and FSC-A = area of the side and forward light scatter pulse. Data from a selected experiment.

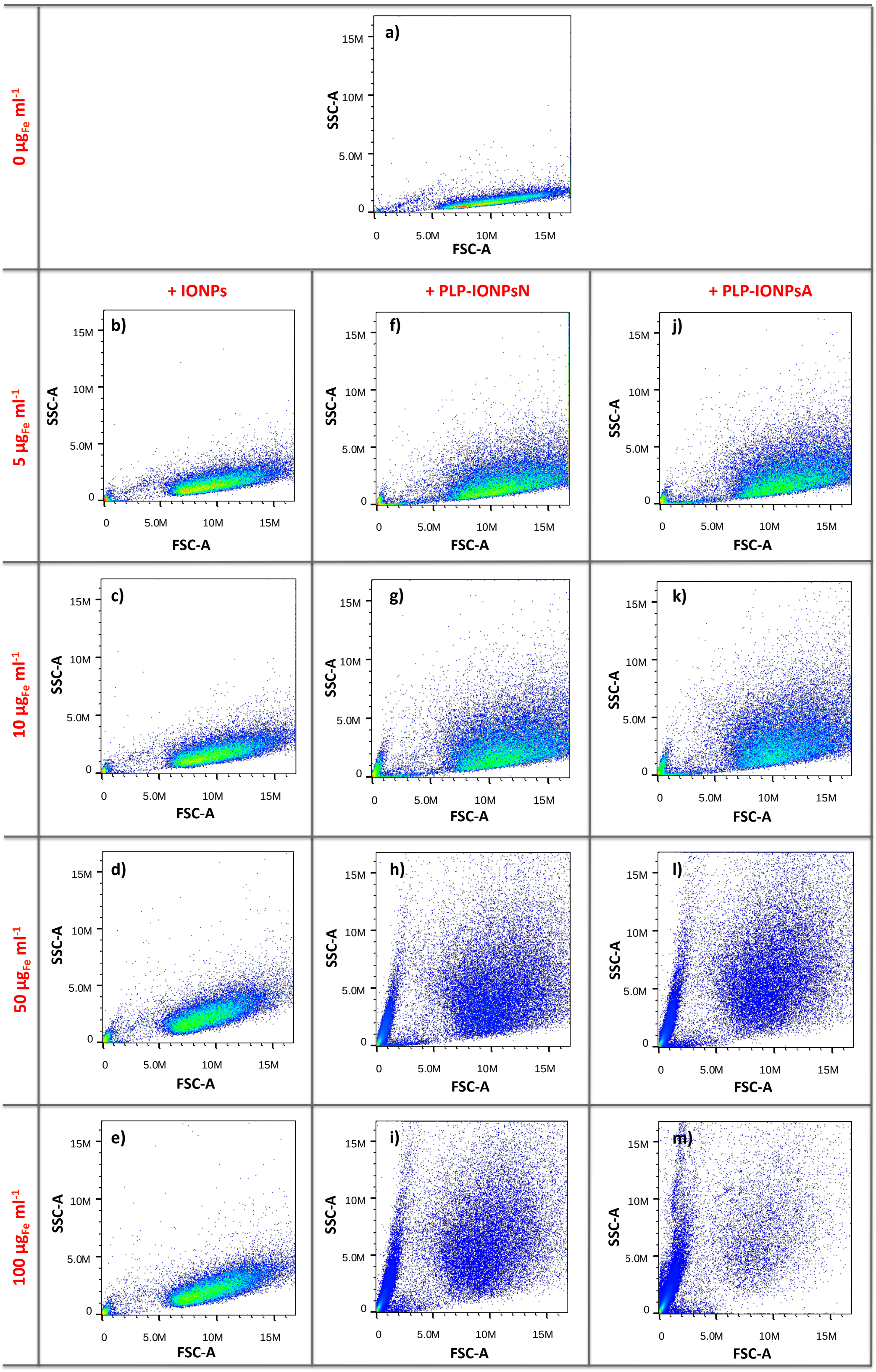

Fig. S6: Forward scatter (FSC) *vs* side scatter (SSC) plot of PC3 cells without IONPs (0 μg$_{Fe}$ ml^{-1}; a) or treated for 24 h with different concentrations (5, 10, 50 and 100 μg$_{Fe}$ ml^{-1}) of uncoated IONPs (b, c, d, e), PLP-IONPsN (f, g, h, i) or PLP-IONPsA (j, k, l, m). SSC-A and FSC-A = area of the side and forward light scatter pulse. Data from a selected experiment.

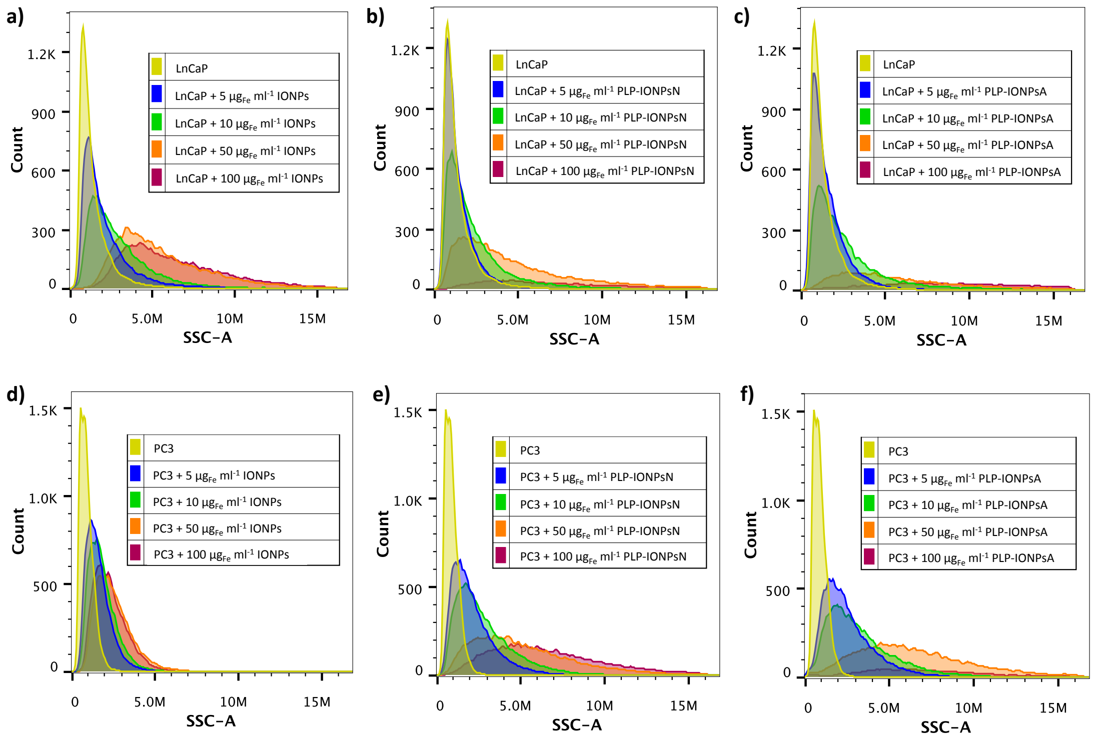

Fig. S7: Counts *vs* area of the side scatter (SSC-A) plot of LnCaP (a, b, c) and PC3 (d, e, f) cells treated for 24 h with different concentrations (5, 10, 50 and 100 μg_{Fe} ml^{-1}) of uncoated IONPs (a, d), PLP-IONPsN (b, e) and PLP-IONPsA (c, f). Data from experiments selected in Fig. S4 and S5.

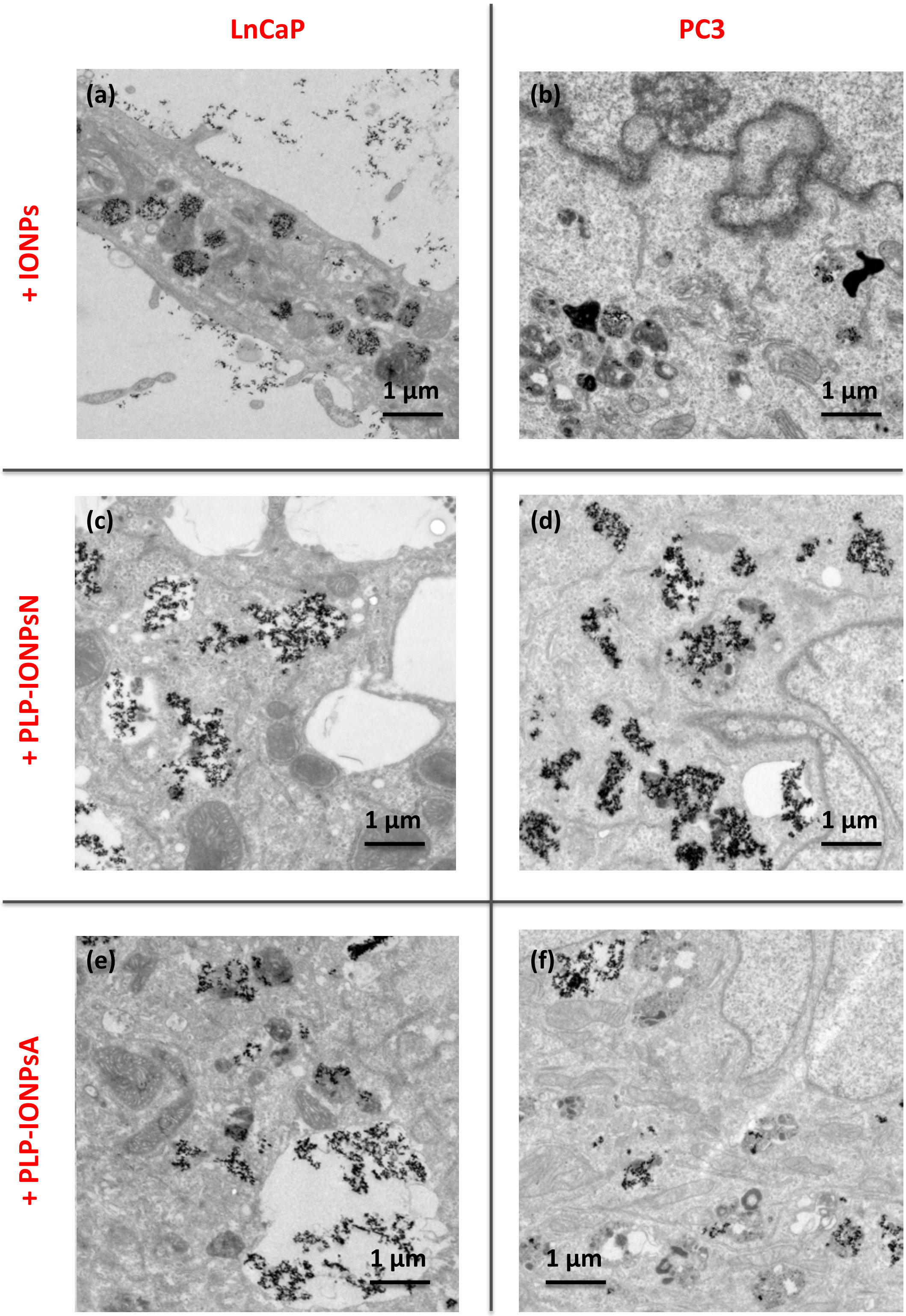

Fig. S8: Representative TEM micrographs of 50 nm-thick cross-sections of LnCaP cells (a, c, e) and PC3 cells (b, d, f) incubated with uncoated IONPs (a, b), PLP-IONPsN (c, d) and PLP-IONPsA (e, f) and embedded in resin.
